# Nursing interventions using digital technologies on individuals with spinal cord injuries: an integrative review

**DOI:** 10.3389/fmed.2026.1912832

**Published:** 2026-08-12

**Authors:** Joana Bernardo Loureiro, Rosa Martins, Ana Francisca Melo, Daniel Ferreira, Emanuel Caramelo, Inês Pereira, Nélia Carvalho, Ricardo Loureiro

**Affiliations:** 1 Center for Innovative Care and Health Technology (ciTechcare), School of Health Sciences, Polytechnic of Leiria, Leiria, Portugal; 2 Jean Piaget Higher School of Health of Viseu, Piaget Institute, Viseu, Portugal; 3 ULS Viseu Dão-Lafões, USF-Montemuro, Viseu, Portugal

**Keywords:** digital health, integrative review, nursing care, rehabilitation, spinal cord injury

## Abstract

**Introduction:**

Spinal cord injury (SCI) is a major global health challenge associated with significant physical, psychosocial, and economic consequences. Digital health technologies have increasingly been integrated into nursing care to support rehabilitation, improve autonomy, and enhance quality of life in individuals with SCI. This integrative review aimed to analyze the impact of nursing interventions using digital technologies in people with SCI.

**Materials and methods:**

An integrative literature review was conducted following the framework of Whittemore and Knafl and reported according to PRISMA 2020 guidelines. A systematic search was performed in MEDLINE (via PubMed), SciELO, and Google Scholar for studies published between 2014 and 2024. Inclusion criteria comprised original empirical studies involving nursing interventions supported by digital technologies in individuals with SCI. Data extraction and analysis focused on intervention characteristics, outcomes, and levels of evidence.

**Results:**

A total of 351 records were identified, and three studies met the inclusion criteria. The studies evaluated multimedia educational resources, telehealth interventions, and a WeChat-based nursing platform. Reported outcomes included improvements in self-perception, functional independence, self-care, health-promoting behaviors, and family functioning. However, the evidence was limited by small sample sizes, heterogeneous methodologies, and short follow-up periods.

**Conclusions:**

The available evidence on digital nursing interventions for individuals with SCI is limited and heterogeneous. The three included studies reported favorable changes in selected rehabilitation and self-management outcomes; however, their methodological limitations and restricted geographical scope preclude definitive conclusions regarding effectiveness or routine implementation. These findings should be interpreted as exploratory, and further rigorous, multicenter, and geographically diverse studies are required.

## 1 Introduction

Spinal cord injury (SCI) is recognized by the World Health Organization (WHO) as one of the most devastating health conditions worldwide, with profound implications for health systems, families, and society (1). The WHO estimates that between 250,000 and 500,000 new cases occur annually and that over 15 million people worldwide live with SCI, although figures may be underestimated in low- and middle-income countries due to weak surveillance systems (1, 2).

Recent evidence reinforces the scale of the problem. A systematic review and meta-analysis by Lu et al. (2) reported a pooled global incidence of 23.77 cases per million people per year (95% CI: 21.50–26.15), with traumatic SCI (TSCI) accounting for 26.48/million and non-traumatic SCI (NTSCI) for 17.93/million. TSCI predominantly affects men, with a male-to-female ratio of about 3.2:1, and is more frequent in young adults aged 21–40 years, whereas NTSCI is increasingly common in older adults, particularly those aged ≥60 years (2). Cervical injuries are the most prevalent in TSCI and often lead to tetraplegia, while NTSCI tends to present with less severe neurological impairment (2, 3).

SCI results from both traumatic causes—such as road traffic accidents, falls, sports injuries, and acts of violence—and non-traumatic causes, including tumors, infections, vascular disorders, and degenerative diseases. Depending on lesion level and severity, individuals may develop paraplegia or tetraplegia, associated with substantial motor, sensory, and autonomic dysfunction (1, 3).

Beyond the immediate neurological damage, individuals with SCI face multiple secondary health conditions, such as urinary tract infections, respiratory complications, pressure ulcers, spasticity, chronic pain, and cardiovascular problems (4). These complications compromise functional independence, increase healthcare needs, and contribute to premature mortality. The WHO reports that people with SCI have two to five times higher risk of dying prematurely, particularly in low-resource settings where access to acute care and rehabilitation is scarce (1).

The psychosocial burden of SCI is equally significant. Depression, anxiety, stigma, and social isolation are frequent, and difficulties with employment, education, and community reintegration further affect quality of life (5). Families also face substantial challenges, as caregiving responsibilities often require financial and emotional adaptations. A systematic review on the economic and quality of life impact of SCI confirmed that individuals face not only high direct healthcare costs (hospitalization, rehabilitation, assistive devices) but also indirect costs related to productivity loss and caregiver dependence, with long-term negative effects on households and health systems (6).

The International Spinal Cord Society (ISCoS) emphasizes that accessible, continuous, and multidisciplinary rehabilitation is fundamental for preventing complications and promoting reintegration (7). Nurses play a pivotal role in this process, intervening in bladder and bowel training, skin integrity, respiratory management, pain control, psychosocial support, and health education. Nursing care extends beyond acute settings, supporting people in the community and at home, and is crucial for maintaining autonomy and quality of life (8).

In recent years, the integration of digital health technologies—such as mobile health (mHealth) applications, teleconsultations, wearable devices, and online educational resources—has gained increasing attention in SCI rehabilitation. These tools facilitate remote monitoring, enhance communication between patients and healthcare professionals, enable early detection of complications, and improve adherence to treatment and rehabilitation programs (9). For individuals with SCI, digital solutions can reduce unnecessary hospital visits, provide health education, and foster greater independence. For nurses, they offer valuable data to support decision-making and proactive interventions (9, 10).

Evidence from recent studies supports the relevance of these innovations. A narrative review highlighted that telehealth and teleSCI programs are feasible, improve continuity of care, and reduce geographic and physical barriers (11). In low- and middle-income countries, telerehabilitation programs have shown particular potential in extending care where resources are limited (12). A systematic review and network meta-analysis further confirmed that telemedicine can prevent and manage pressure injuries in individuals with SCI, reducing both incidence and severity (13). Another recent study demonstrated that digital interventions, including smartphone apps and teleconsultations, can improve quality of life and reduce symptoms of depression in people with SCI (14).

Despite these promising findings, challenges remain. Barriers include disparities in access to internet and devices, limited digital literacy among patients and caregivers, and concerns regarding data security and privacy (15). Moreover, much of the existing evidence is still based on small-scale or descriptive studies, and there is a need for more rigorous trials to assess long-term effectiveness and scalability (2).

Aligned with the WHO *Global Strategy on Digital Health 2020–2025*, strengthening the evidence base in this field is crucial to promote equity, ensure safe implementation, and support health professionals—especially nurses—in adopting innovative approaches (16).

Thus, the aim of this study was to conduct an integrative literature review to explore and analyze the impact of nursing interventions using digital technologies in individuals with spinal cord injury. The specific objectives were: (i) to identify digital tools applied in nursing care for SCI; (ii) to evaluate their reported outcomes in terms of quality of life, functional independence, adherence, and complication prevention; and (iii) to identify gaps and challenges that may inform future research and clinical practice.

## 2 Materials and methods

### 2.1 Type of study

This study adopted the methodology of an Integrative Literature Review (ILR), recognized as one of the broadest types of review, as it allows the inclusion and synthesis of studies with different methodological designs—experimental, quasi-experimental, and descriptive (17). The ILR provides a more comprehensive understanding of complex health phenomena, integrating empirical evidence with theoretical perspectives. The methodological steps were based on the framework of Whittemore and Knafl (17) and adapted to ensure transparency and rigor following the PRISMA 2020 recommendations (18). The process was developed in six sequential stages: (i) problem identification, (ii) definition of eligibility criteria, (iii) systematic literature search, (iv) screening and selection of studies, (v) data extraction, and (vi) synthesis and analysis of results.

### 2.2 Research question

The guiding question was developed using the **PICO strategy**, which provides a systematic structure for framing research problems in health sciences:

**Population (P):** Individuals with spinal cord injury (SCI).

**Intervention (I):** Nursing interventions supported by digital health technologies.

**Comparison (C):** Conventional care or absence of digital interventions.

**Outcomes (O):** Quality of life, functional independence, complication prevention, treatment adherence, and psychosocial wellbeing.

The formulated research question was: “What is the impact of nursing interventions using digital health technologies on the outcomes of individuals with spinal cord injury?”

This formulation aimed to ensure clarity, reduce ambiguity, and facilitate the identification of relevant keywords and descriptors for database searches.

### 2.3 Inclusion and exclusion criteria

The eligibility criteria were established a priori to ensure the methodological consistency of the review.

Inclusion criteria: original empirical studies (quantitative, qualitative or mixed methods) involving people with SCI; nursing interventions mediated by digital technologies (e.g., mHealth apps, telehealth, wearables, multimedia education); outcomes on function, quality of life, ADLs/independence, adherence, prevention of secondary complications, or psychosocial aspects; publications between Jan-2014 and Nov-2024 in English, Portuguese or Spanish.

Exclusion criteria: reviews, editorials, commentaries, conference abstracts, and studies not directly involving nursing interventions or not reporting outcomes. Access limitations were not used as an a priori exclusion criterion; when full texts could not be retrieved after reasonable attempts, these records were reported in the PRISMA flow as “not retrieved.”

The chosen timeframe (2014–2024) was justified by the significant global growth of digital health solutions in the last decade, particularly in the field of chronic and rehabilitative care.

### 2.4 Search strategy

The literature search was conducted in 08/11/2024 in two databases and one gray literature chosen for their coverage and relevance to health and nursing research:

- MEDLINE (via PubMed) – main biomedical database with indexed scientific articles.
- SciELO (Scientific Electronic Library Online) – ensures access to publications from Portuguese- and Spanish-speaking countries.
- Google Scholar – to broaden coverage and capture gray literature not indexed in traditional databases.

MEDLINE via PubMed was selected because of its broad coverage of biomedical and health sciences research. SciELO was included to enhance the identification of literature from Portuguese- and Spanish-speaking countries, while Google Scholar was used as a supplementary information source to broaden retrieval beyond conventionally indexed journal literature.

A three-step search process was used to identify relevant published studies. First, a preliminary limited search was conducted in MEDLINE via PubMed to identify articles related to nursing interventions supported by digital technologies for individuals with spinal cord injury. The text words contained in the titles and abstracts of potentially relevant articles, together with the index terms used to describe these articles, were analyzed to identify appropriate keywords, synonyms, and controlled vocabulary terms, including Medical Subject Headings (MeSH). Second, the identified text words, keywords, synonyms, and index terms were combined to develop the complete search strategy. This strategy was subsequently adapted to the indexing system, search interface, and technical characteristics of each information source included in the review: MEDLINE via PubMed, SciELO, and Google Scholar. Third, the reference lists of the studies included in the review were screened to identify any additional potentially eligible studies.

The final search expressions differed across the three information sources. MEDLINE via PubMed used a structured combination of terms related to spinal cord injury, nursing and digital health interventions, and selected outcomes. Broader combinations were used in SciELO and Google Scholar, reflecting the more limited indexing and search functionalities of these sources. The exact strategy applied to each information source, without retrospective modification, is presented in Table 1. Boolean operators were used to refine the search results, and all searches were conducted in Portuguese, English, and Spanish, whenever possible, to broaden the scope of the evidence retrieved.

**Table 1:** Search strategies applied in scientific databases.

Search strategy	Gray literature resource/ scientific database	Number of records retrieved
Spinal Cord Injuries AND Nursing AND Digital	Google Scholar	100
Spinal Cord Injuries and nursing	SciELO	27
(“Spinal Cord Injuries”[MeSH Terms] OR “Spinal Cord Injury”[Title/Abstract] OR “Spinal Shock”[Title/Abstract] OR “Paraplegia”[MeSH Terms] OR “Paraplegia”[Title/Abstract] OR “Tetraplegia”[Title/Abstract]) AND (“Nursing”[MeSH Terms] OR “Nursing”[Title/Abstract] OR “Telenursing”[MeSH Terms] OR “Telenursing”[Title/Abstract] OR “Digital Health”[MeSH Terms] OR “Digital Health”[Title/Abstract] OR “eHealth”[Title/Abstract] OR “mHealth”[Title/Abstract] OR “Telemedicine”[MeSH Terms] OR “Telemedicine”[Title/Abstract]) AND (“Quality of Life”[MeSH Terms] OR “Quality of Life”[Title/Abstract] OR “Functional Independence”[Title/Abstract] OR “Activities of Daily Living”[MeSH Terms] OR “Activities of Daily Living”[Title/Abstract])	MEDLINE (via PubMed)	224

### 2.5 Data extraction and analysis

The data extracted from the selected studies were organized and analyzed systematically, according to the following steps:

Step 1: Data Extraction and Organization: Relevant information from each study was extracted using a structured form (Table 2), which included the following fields: author(s), year and country of publication, title, study objective, study design, participants, intervention and data collection instruments, results, and level of evidence (19). Each study was appraised independently by two reviewers, and discrepancies were resolved by consensus. A summary of the quality assessment criteria and results is presented in supplementary material. The extracted information was then organized in synthesis tables to facilitate comparison across studies and to identify patterns or discrepancies in the findings.

**Table 2:** Data extraction and organization table.

Field	Description
Study authors; year of publication and country	Name(s) of the study authors; Year of publication and country where the study was conducted
Study design; aim; sample	Experimental, quasi-experimental, descriptive, or cross-sectional studies; Description of the study's objective
Intervention and comparator	Description of the intervention and data collection instruments
Main findings	Summary of the main findings
Effect size/effect estimate	Description of effect size/effect estimate

Step 2: Data Analysis and Synthesis: Following data extraction, the included studies were compared across intervention modality, technological function, degree of interactivity and personalization, nursing activities, duration and continuity of contact, outcome domains, follow-up periods, and methodological quality. An iterative comparison was undertaken to identify recurrent patterns, differences, and conceptual relationships across the studies. The findings were organized into three cross-cutting domains: (i) digitally mediated education and self-management support; (ii) continuity, interaction, and relational nursing support; and (iii) functional, clinical, behavioral, and family-related outcomes. Methodological differences and contradictory or non-significant findings were retained in the synthesis rather than subsumed within an overall positive interpretation. Given the substantial heterogeneity and small number of studies, the findings were synthesized narratively and were not quantitatively pooled.

Step 3: Presentation of Results: The results were organized and presented in tables and descriptive narratives, synthesizing the evidence on digital nursing interventions in SCI. This presentation supports the understanding of the available evidence and contributes to informed decision-making in nursing practice focused on rehabilitation, autonomy, and complication prevention in individuals with spinal cord injury.

The review process was reported in accordance with the PRISMA 2020 checklist (18), which is provided as a supplementary file.

## 3 Results

### 3.1 Study selection

The initial search identified 351 records (PubMed = 224; SciELO = 27; Google Scholar = 100). After removing 7 duplicates, 344 records were screened by title/abstract and 142 were excluded. Two hundred two full texts were sought for retrieval; 146 could not be obtained (duplicate indexing/access restrictions). Fifty-six full texts were assessed for eligibility; 53 were excluded (49 due to ineligible population/intervention; 3 due to ineligible population). Finally, 3 studies (S1–S3) met all inclusion criteria and were included in the final synthesis. The selection process followed the PRISMA 2020 flow diagram (18).

Google Scholar was searched on using the expression “Spinal Cord Injuries AND Nursing AND Digital.” Results were ordered by relevance, and the first 100 results returned by the platform were screened. As no a priori stopping criterion had been established, the search was limited to these first 100 results. References considered potentially relevant were manually recorded and subsequently combined with the records retrieved from MEDLINE via PubMed and SciELO. Duplicate records were identified through comparison of titles, authors, publication years, and digital object identifiers, when available, and were removed before screening.

**Figure 1:**
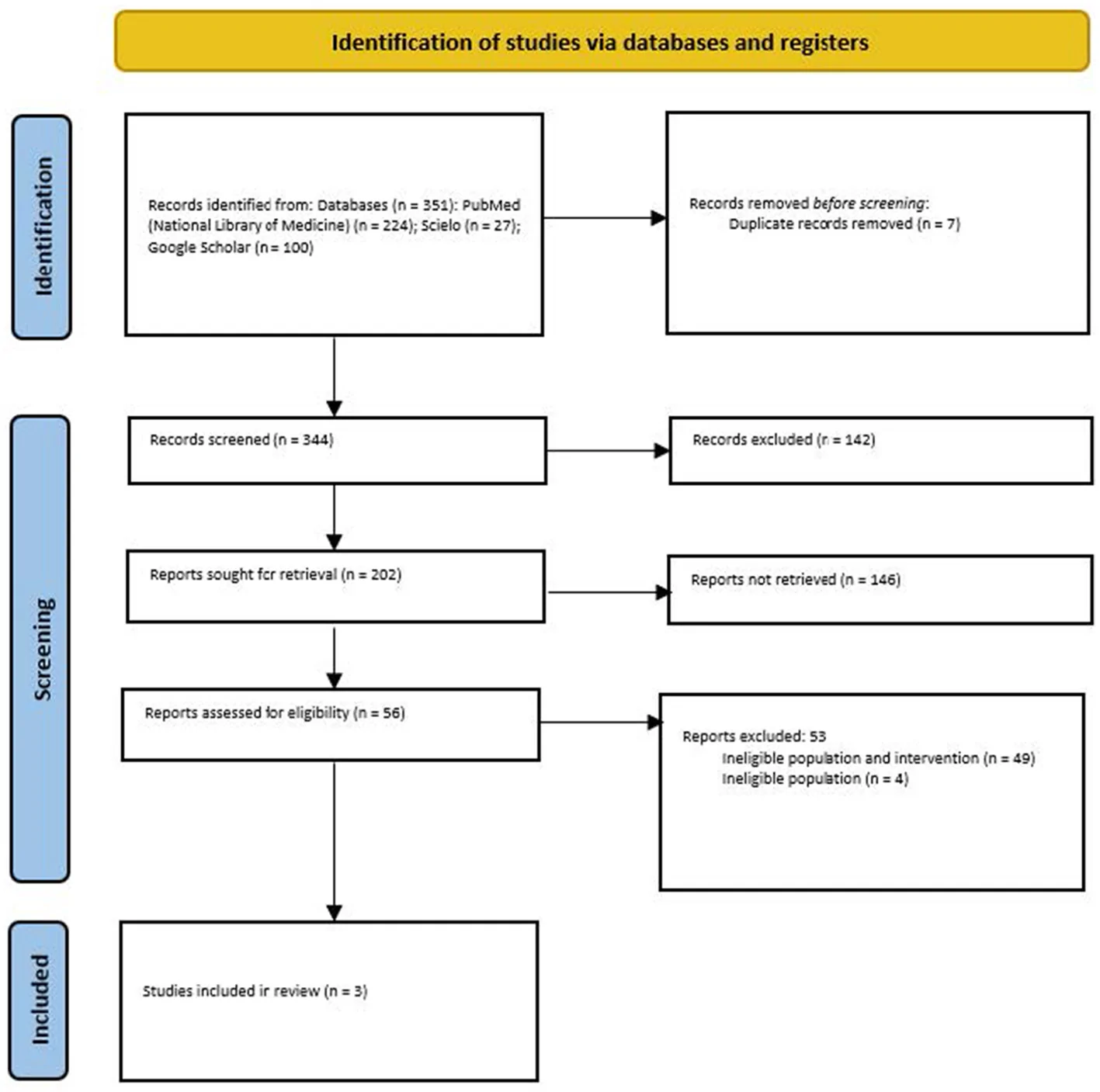
PRISMA 2020 flow diagram of study selection process (18).

The study selection process is illustrated in the PRISMA 2020 flow diagram (Figure 1).

### 3.2 Characteristics of included studies

The included studies (2015–2021, all in Asia) enrolled samples ranging from 2 to 78 participants and evaluated a multimedia DVD, a nurse-led telehealth programme, and a WeChat-based continuous nursing model. The interventions included:

- a multimedia DVD program for home-based rehabilitation (in Taiwan);
- a telehealth program combining phone calls, video consultations, and WhatsApp (in India);
- a mobile communication platform (WeChat) to support continuous nursing care at home (in China).

The studies addressed populations ranging from 2 to 78 participants, including individuals post-discharge with SCI. The main characteristics of the included studies are presented in Table 3.

**Table 3:** Summary of studies included in the integrative review.

Study authors; year of publication and country	Study design; aim; sample	Intervention and comparator	Outcomes	Instruments	Follow-up	Main findings	Effect size/effect estimate
Chen et al. (20); Taiwan	Quasi-experimental pretest–posttest study; To assess the effects of a spinal cord injury home rehabilitation DVD on patients with spinal cord injury; *n* = 59: intervention *n* = 28, control *n* = 31; age NR; both sexes	Home rehabilitation multimedia DVD containing rehabilitation exercises and self-care information; control condition: conventional care/education	Self-perception; self-efficacy	For the self-perception scale, the eight item scale proposed by (26) was adopted. This scale was administered to 210 patients, producing a Cronbach's α of 0.87. For the self-efficacy scale, the approved Chinese version of the Moorong Self-Efficacy Scale, originally developed by (28) and translated by (27), was adopted. This scale was administered to 210 patients, producing a Cronbach's α of 0.90. Both scales adopted a five-point scoring system, wherein a high score indicated high self-perception or self-efficacy toward the disease.	Pre-intervention and immediate post-intervention; no long-term follow-up reported	Greater improvement in self-perception in the intervention group; self-efficacy increased in both groups, but no significant between-group difference was identified	Based on the power analysis for a two-group independent sample test, with a power of 0.8 and an effect size of 0.8, the sample size of the experimental group was approximately 26, and that of the control group was approximately 26. To avoid losing any participants, the sample sizes of 28 and 31 were selected for the experimental and control groups respectively.
Tyagi et al. (21); India	Case series; to provide supervision and guidance to two individuals with SCIs through the use of telehealth; *n* = 2: one 44-year-old man with C3 AIS C SCI and one 35-year-old woman with T12 AIS A SCI	Nurse-supported telehealth using telephone calls, live video communication, WhatsApp, caregiver training, and individualized home goals; no comparator	Functional independence in self-care and mobility	Self-reported Spinal Cord Independence Measure III — SCIM III	Biweekly monitoring over **4 weeks**, followed by post-intervention assessment	Self-care increased from 3 to 15 and from 4 to 15; mobility increased from 14 to 27 and from 4 to 16 in the two participants, respectively	Standardized effect size not applicable; absolute self-care changes: +12 and +11; mobility changes: +13 and +12.
Li et al. (22); China	Randomized controlled trial; to analyse the effect of participatory continuous nursing using the WeChat platform on the complications, family function and compliance of patients with spinal cord injuries; *n* = 78: intervention *n* = 39, control *n* = 39; mean age 47.12 ± 10.90 years; 40 men and 38 women	Six-month participatory continuous nursing through WeChat vs. regular post-discharge care	Secondary complications; family function; health-promoting behaviors	Clinical recording of complications; Family Assessment Device – FAD. The FAD comprises seven dimensions: problem solving, communication, role function, emotional response, emotional intervention, behavior control and total function; modified Health-Promoting Lifestyle Profile II - HPLP-II. Items on the HPLPII are scored using a four-point Likert scale: 1 = never, 2 = sometimes, 3 = often, 4 = the same. The total possible score ranges from 52 to 208. Higher scores indicate better health.	Baseline, 3 months, and 6 months after discharge	Lower rates of pressure injuries, urinary tract infections, joint contractures, and muscle atrophy; better family function and health-promoting behaviors at 3 and 6 months after discharge	Patients with stable disease treated by internal fixation between August 2017 and August 2019 in a major tertiary teaching hospital in China were recruited using convenience sampling.

AIS, American Spinal Injury Association Impairment Scale; FAD, Family Assessment Device; HPLP-II, Health-Promoting Lifestyle Profile II; MD, mean difference; NR, not reported; RR, risk ratio; SCI, spinal cord injury; SCIM III, Spinal Cord Independence Measure III. Effect sizes were calculated using the summary data reported in the original study. For FAD, lower scores indicate better family functioning; therefore, the negative effect size favors the intervention group.

Across the three included studies, digital technology did not operate as an independent therapeutic component, but primarily as a means of delivering nursing education, individualized guidance, follow-up, and self-management support beyond the institutional setting. The interventions could be positioned along a tentative continuum of increasing interactivity and continuity. The multimedia DVD provided standardized, unidirectional educational content; the telehealth intervention enabled bidirectional communication, individualized goal-setting, and caregiver involvement; and the WeChat intervention supported continuous communication, health education, monitoring, and family-oriented nursing guidance over 6 months.

Three interrelated outcome domains were identified. First, educational and self-management-related outcomes included self-perception, self-efficacy, and health-promoting behaviors. Second, functional outcomes included self-care, mobility, and independence in activities of daily living. Third, clinical and relational outcomes included secondary complications and family functioning. The intervention using a static educational resource was primarily associated with a proximal cognitive outcome—self-perception—without a significant between-group effect on self-efficacy. The more interactive and sustained interventions reported changes across broader functional, clinical, behavioral, and family-related domains.

This pattern may suggest that interactivity, personalization, continuity of nursing contact, and integration of family support are potentially relevant intervention components. Nevertheless, this relationship should be considered hypothesis-generating only. The interventions differed simultaneously in technological modality, nursing intensity, duration, study design, sample size, comparator conditions, and outcome measurement. Therefore, the review cannot determine whether the reported differences were attributable to the technology itself, the nursing support delivered through it, intervention duration, contextual factors, or methodological variation.

The three included studies (S1–S3) highlight the potential of digital health tools in complementing nursing interventions for individuals with spinal cord injury, although with varying levels of effectiveness and important limitations.

#### 3.2.1 S1—home-based rehabilitation (multimedia DVD)

The quasi-experimental study demonstrated statistically significant improvements in self-perception among participants in the intervention group, with mean scores increasing from 30.18 to 39.96 (*p* < 0.05). However, changes in self-efficacy were not statistically significant (*p* > 0.05). While the DVD proved effective as an educational tool for reinforcing home-based rehabilitation, its impact was limited by the lack of interactivity and personalization, reducing patient engagement and adaptation to individual needs.

#### 3.2.2 S2—telehealth (phone calls, video calls, and WhatsApp)

The telehealth intervention showed promising results in improving activities of daily living (ADLs), with participants reporting an average 60% increase in SCIM III scores. In addition, the program significantly reduced financial costs associated with hospital visits and transportation, demonstrating the economic feasibility of digital follow-up. Despite these positive outcomes, the very small sample size (*n* = 2) limits the generalizability of the findings and calls for larger, more robuststudies.

#### 3.2.3 S3—digital platform (WeChat)

The study assessed the impact of continuous nursing follow-up using the WeChat platform. The intervention was effective in reducing complications, with pressure ulcers decreasing from 23.08% to 5.13% and urinary tract infections from 20.51% to 2.56% (*p* < 0.05). Furthermore, significant improvements were reported in family function (FAD scores decreased from 128.92 to 113.67, *p* < 0.001) and health-promoting behaviors (HPLPII scores increased from 122.26 to 138.69, *p* < 0.001). These findings highlight the potential of mobile platforms in facilitating ongoing communication, health education, and adherence to rehabilitation programs. However, their applicability depends heavily on digital literacy and access to mobile devices, which may exclude vulnerablepopulations.

## 4 Discussion

This integrative review analyzed three studies (S1–S3) that explored the use of digital health technologies in nursing interventions for individuals with spinal cord injury (SCI). Overall, the findings suggest that digital tools such as multimedia educational resources, telehealth, and mobile platforms can effectively complement traditional rehabilitation and nursing care. These technologies were associated with improvements in self-perception, autonomy in activities of daily living, prevention of complications, and psychosocial outcomes.

Rather than demonstrating the effectiveness of a specific technology, the findings indicate that digital tools were used as delivery mechanisms for three interconnected nursing functions: health education and self-management support, continuity of care after discharge, and monitoring or prevention of secondary complications. The reported outcomes should therefore be interpreted in relation to the nursing intervention delivered through the technology, rather than attributing effects to the technological platform alone.

The first study (S1), which evaluated the use of a DVD-based educational program for home rehabilitation, demonstrated significant improvements in self-perception (*p* < 0.05) but no statistically significant changes in self-efficacy (20). This finding indicates that structured educational tools can strengthen patients' awareness and confidence in managing their condition, although their impact on behavioral change appears limited. Such limitations are likely linked to the static and non-interactive nature of the resource, which does not allow personalization or real-time feedback. This result is consistent with research emphasizing that interactive and tailored digital health tools are more effective in promoting long-term engagement and adherence (8, 15). In this sense, the WHO's *Global Strategy on Digital Health 2020–2025* highlights that digital interventions must be centered on users' needs and adaptable to different contexts to maximize their impact (16, 23).

The limited interactivity and personalization identified in S1 should also be considered in light of more recent SCI-specific mobile application development studies published after the completion of the present review search. Daeechini et al. (24) identified educational information, disease and complication management, patient profile, and technical capabilities as core requirements for an SCI self-management mobile application, based on the perspectives of both individuals with SCI and relevant specialists. In a subsequent developmental study (25), a Persian-language self-management application that included modules addressing autonomic dysreflexia, skin care, bowel and bladder management, lifestyle, and access to specialist support demonstrated acceptable short-term usability among individuals with SCI. These findings reinforce that the potential value of digital interventions depends not merely on the use of technology, but on user-centered development, clinically relevant functionalities, accessible interfaces, and adaptation to users' linguistic, cultural, and healthcare contexts. (24, 25). Nevertheless, as these studies primarily addressed requirements, development, and short-term usability, further research is required to determine whether such applications produce sustained improvements in self-management, complication prevention, functional outcomes, or quality of life.

The second study (S2), conducted in India, explored the use of telehealth through phone calls, video consultations, and WhatsApp messaging. This intervention was associated with improvements in activities of daily living, with participants reporting an average 60% increase in SCIM III scores, as well as a reduction in healthcare-related costs (21). These findings underscore the potential of telehealth to provide continuity of care after hospital discharge, overcoming barriers related to distance, cost, and mobility. This is particularly relevant in low-resource settings, where access to specialized rehabilitation services is often limited. These results are in line with international guidelines, such as those from ISCoS, which emphasize that continuity of care and long-term rehabilitation are essential to prevent complications and promote autonomy (7). Furthermore, systematic reviews indicate that telehealth can improve quality of life and reduce complications in SCI, although most available studies remain small-scale or exploratory (9, 12, 13). The reduced sample size of S2 (*n* = 2) illustrates this limitation, highlighting the urgent need for larger and more robust trials to confirm these promising outcomes.

The third study (S3), conducted in China, offered the most comprehensive evidence by analyzing the use of the WeChat platform for continuous nursing care. The intervention demonstrated significant reductions in complications, with pressure ulcers decreasing from 23.08% to 5.13% and urinary tract infections from 20.51% to 2.56% (*p* < 0.05). Additionally, there were improvements in family function (*p* < 0.001) and health-promoting behaviors (*p* < 0.001), showing that digital platforms can extend the scope of nursing interventions beyond clinical management to include psychosocial and family dimensions of care (22). These findings reinforce the notion that mobile health interventions can be effective in supporting adherence, enhancing communication, and preventing complications (9, 13, 14). However, as other studies have pointed out, the success of such interventions depends heavily on patients' digital literacy and access to technology, which may limit their applicability in vulnerable populations, such as older adults or those living in rural areas (15).

Taken together, the findings from S1–S3 reinforce the central role of nursing in integrating digital health technologies into SCI rehabilitation. Nurses are positioned at the frontline of patient education, complication prevention, and long-term follow-up, and digital tools offer new opportunities to enhance these roles. By leveraging technology, nurses can provide continuous guidance, monitor patients remotely, and engage families in the care process, thereby strengthening self-management and autonomy. Nonetheless, the success of these interventions depends on appropriate training for professionals, careful adaptation to patient needs, and strategies to reduce barriers to digital inclusion (7, 15, 23).

Despite these promising results, several limitations must be acknowledged. First, only three studies met the eligibility criteria, and their designs, sample sizes, intervention modalities, measurement instruments, outcomes, and follow-up periods were substantially heterogeneous. This heterogeneity prevented direct comparison and did not allow the findings to be quantitatively pooled. Second, the included studies were conducted exclusively in Asian countries, limiting the transferability of the findings to other geographical, cultural, linguistic, socioeconomic, and healthcare contexts. Third, the small samples—particularly the case study involving only two participants—and the limited follow-up periods reduce confidence in the precision and sustainability of the reported outcomes. These methodological limitations reflect broader gaps in the literature, as noted in recent reviews, which call for high-quality randomized controlled trials, standardized outcome measures, and multicenter studies involving larger and more diverse populations (9, 12–14).

The methodological scope of the review should also be acknowledged. The search was limited to MEDLINE via PubMed, SciELO, and Google Scholar and to publications in English, Portuguese, and Spanish, which may have resulted in relevant studies indexed in other databases or published in other languages being missed. The conceptual structure of the search strategy was not fully equivalent across the included information sources. The MEDLINE via PubMed strategy incorporated an additional outcome-related concept that was not consistently included in the SciELO and Google Scholar searches. Conversely, the broader strategies used in SciELO and Google Scholar may have retrieved records with a different conceptual scope. These differences may have affected search sensitivity, specificity, reproducibility, and comprehensiveness and may have contributed to relevant studies being missed. The possibility of publication and selection bias cannot be excluded and could not be formally assessed because of the small number of included studies. Moreover, the methodological limitations and variable levels of evidence of the primary studies constrain the strength of the synthesis. Therefore, this review should be interpreted as exploratory and hypothesis-generating rather than confirmatory. Its findings identify preliminary patterns and research gaps but do not establish the effectiveness of digital nursing interventions for people with spinal cord injury.

From a clinical perspective, the findings identify potential areas in which digital technologies may support nursing care, including health education, post-discharge communication, self-management support, and monitoring of secondary complications. However, the available evidence is insufficient to recommend their routine implementation or to determine which technological modalities, intervention components, or delivery models are most effective. Any implementation should therefore be context-sensitive, accompanied by assessment of accessibility and digital literacy, and subject to ongoing evaluation of feasibility, acceptability, safety, and clinical outcomes.

At the policy level, the findings highlight areas requiring further investigation rather than supporting definitive policy recommendations. Future studies should examine infrastructure requirements, professional training, digital inclusion, data protection, and cost-effectiveness before these interventions are incorporated into standard SCI rehabilitation pathways. Cost-effectiveness analyses are also needed to support decision-makers in evaluating the potential of digital health to reduce long-term healthcare costs associated with SCI (6). As highlighted by the WHO and ISCoS, ensuring accessibility and continuity of care through digital innovation is essential to reduce disparities and strengthen rehabilitation services (1, 7, 16).

In summary, the results of this review indicate that digital nursing interventions have the potential to improve outcomes for people with SCI by enhancing self-management, promoting independence, reducing complications, and supporting psychosocial wellbeing. However, the current evidence is still limited in scope and quality, requiring more robust research to consolidate best practices and inform guidelines. Strengthening the evidence base through rigorous trials, supporting equitable access, and recognizing the central role of nurses in digital innovation are key steps for advancing SCI rehabilitation and improving quality of life for this population.

## 5 Conclusions

This integrative review identified a small and heterogeneous body of evidence regarding nursing interventions supported by digital technologies for individuals with spinal cord injury. Across the three included studies, favorable changes were reported in selected outcomes, including self-perception, functional independence, health-promoting behaviors, family functioning, and the occurrence of some secondary complications. These findings suggest that multimedia education, telehealth, and mobile communication platforms may have a supportive role in specific components of nursing care and rehabilitation.

However, the limited number of studies, small samples, methodological heterogeneity, short or variable follow-up periods, and concentration of all studies in Asian settings preclude firm conclusions regarding effectiveness, sustainability, or generalizability. Accordingly, the findings should be interpreted as exploratory and hypothesis-generating rather than confirmatory and do not currently support strong recommendations for routine clinical implementation. Further rigorously designed, adequately powered, multicenter studies involving geographically and culturally diverse populations are required to determine the effectiveness, safety, acceptability, and cost-effectiveness of digital nursing interventions for people with spinal cord injury.
